# Supplementary material for: Structures of neurexophilin–neurexin complexes reveal a regulatory mechanism of alternative splicing
Source: EMBO J. 2019 Sep 30;38(22):e101603. doi: 10.15252/embj.2019101603 (PMC6856630; doi:10.15252/embj.2019101603)
Supplement: Supplementary file 1 — Appendix [file EMBJ-38-e101603-s001.pdf]

**Appendix**

# Structures of neurexophilin-neurexin complexes reveal a regulatory mechanism of alternative splicing

Steven C. Wilson[1], K. Ian White[1], Qiangjun Zhou[1], Richard A. Pfuetzner[1], Ucheor B. Choi[1], Thomas C. Südhof[1,2], and Axel T. Brunger[1,2]

[1]Department of Molecular and Cellular Physiology, Stanford University Medical School, Stanford, CA 94305, USA

[2]Howard Hughes Medical Institute

## Table of Contents

**Appendix Figure S1** Superposition of structures of uncomplexed LNS2$^{SS2-}$ and Nxph1-complexed LNS2 $^{SS2-}$ ........................................................................ 2

**Appendix Figure S2** Glycosylated and cysteine-rich regions in neurexophilin ............. 3

**Appendix Figure S3** Packing of complexes in Nxph1-LNS2$^{SS2A+}$ crystals and details of Nxph1-LNS2$^{SS2A+}$ interface architecture ........................................................ 4

**Appendix Figure S4** Co-immunoprecipitation of Nrxn3 LNS2$^{SS2-}$ mutants with Nxph1 and Circular Dichroism spectra of LNS2 and Nxph proteins .............................. 5

**Appendix Figure S5** Trace amounts of $Ca^{2+}$ in buffer do not affect the binding affinity of Nxph1 for Nrxn1 LNS2$^{SS2-}$ and Nrxn1 LNS2$^{SS2A+}$ ........................................... 6

**Appendix Figure S6** Tandem affinity purification of Nxph1-Nrxn1 LNS2 complexes........................................................................................................... 7

**Appendix References** .................................................................................. 8

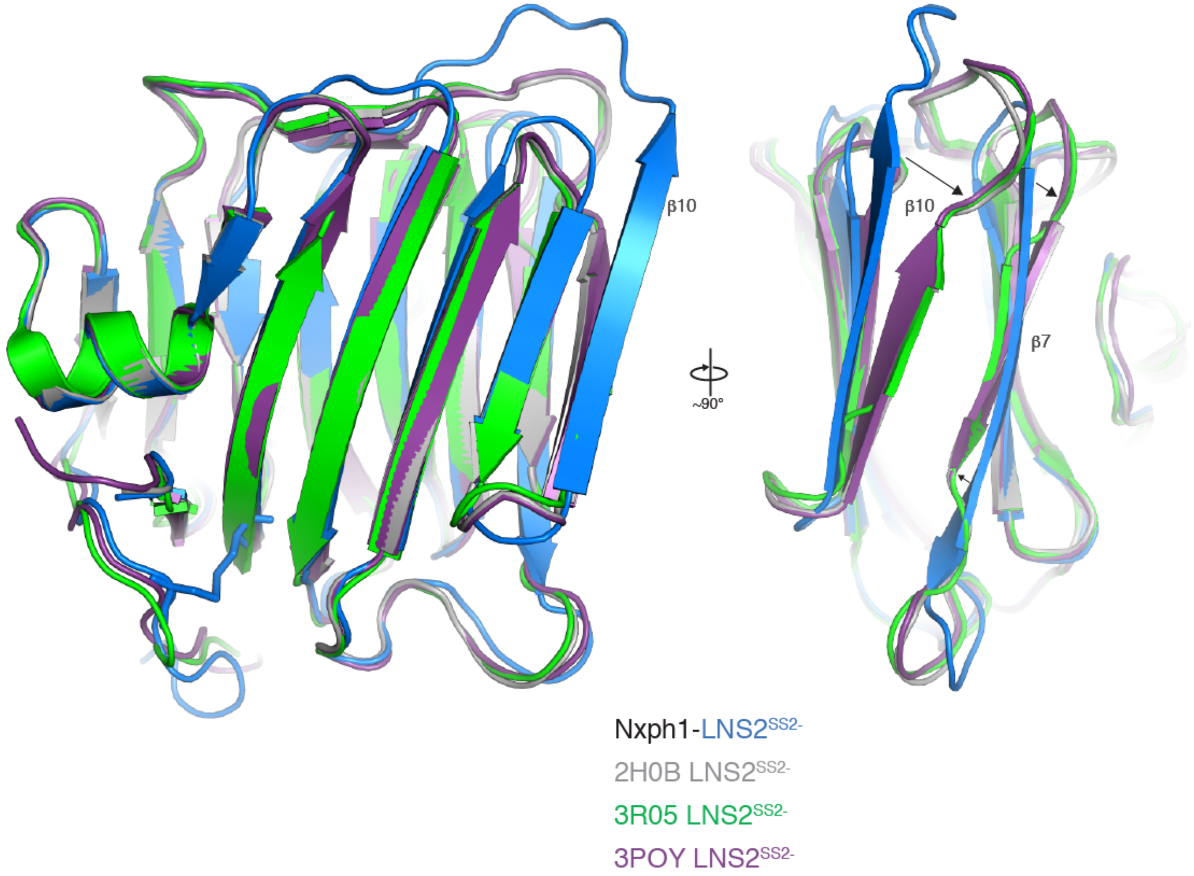


**Appendix Figure S1** Superposition of structures of uncomplexed LNS2$^{SS2-}$ and Nxph1-complexed LNS2 $^{SS2-}$. Aligned LNS2$^{SS2-}$ domains from Nxph1-LNS2$^{SS2-}$, 2H0B, 3POY, and 3R05 structures are shown. Arrows indicate differences between the conformations of β7 and β10, and the loop after β10 in the Nxph1-LNS2$^{SS2-}$ and uncomplexed LNS2$^{SS2-}$ structures.

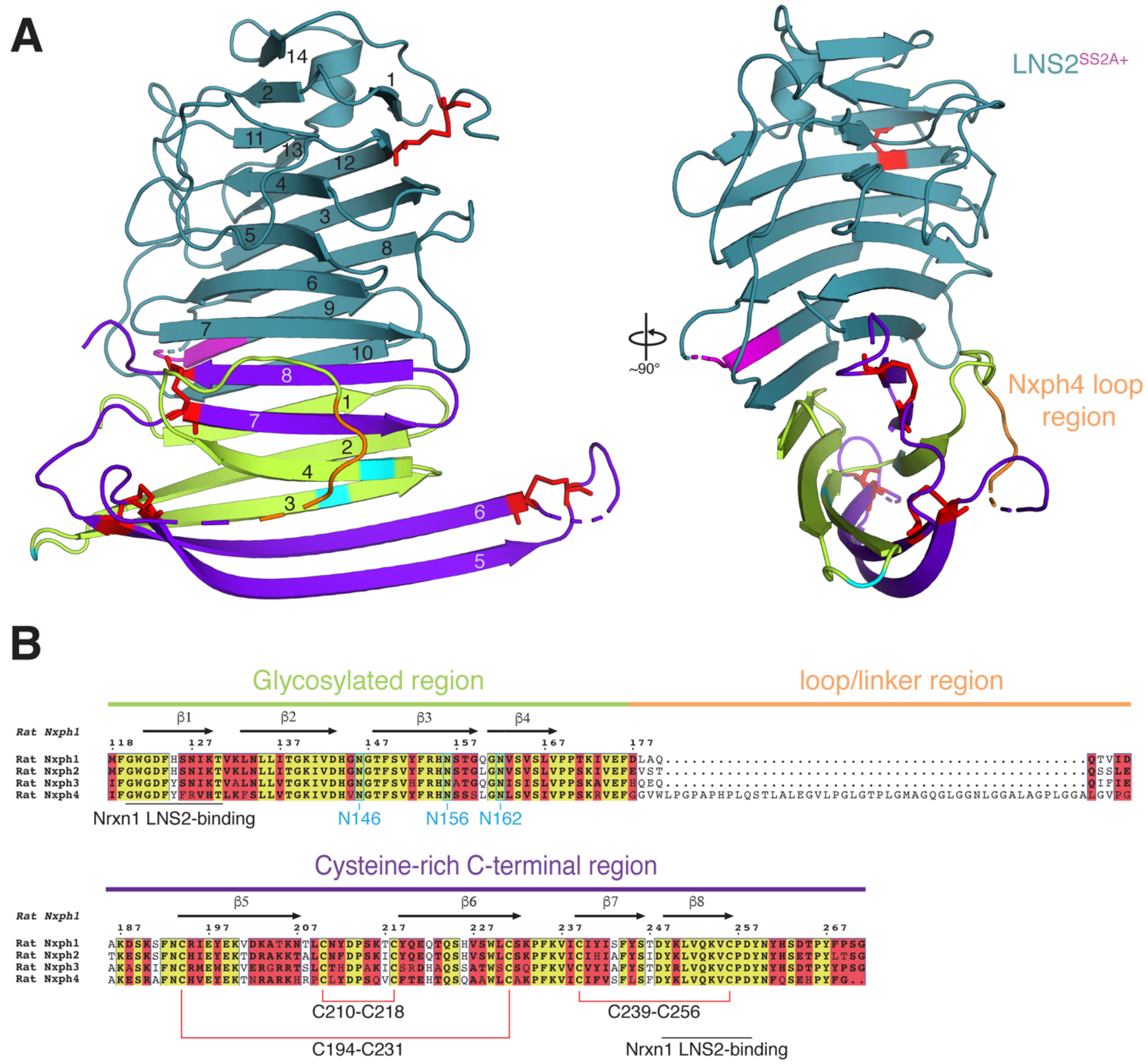


**Appendix Figure S2** Glycosylated and cysteine-rich regions in neurexophilin. (**A**) Views of the Nxph1-LNS2$^{SS2A+}$ structure. The previously proposed neurexophilin N- and C-terminal domains (Missler & Südhof, 1998) are colored green and purple, and the loop region connecting strands β4 and β5 is colored orange. Disulfides are colored red, and N-linked glycosylation sites are colored blue. The β-strands of LNS2$^{SS2A+}$ and Nxph1 are numbered. The SS2A insert is colored magenta in teal-colored LNS2$^{SS2A+}$. (**B**) Sequence alignment of rat Nxph1-4 with the color-coded regions in **A** are indicated with text and lines. The disulfide bonds are indicated with red connecting lines, and the highly conserved N-linked glycosylation sites in the sequence alignment are outlined with blue boxes. Sequences were aligned using Clustal Omega (Sievers *et al*, 2011), and the secondary structure from the Nxph1 structure is shown at the top of the sequence alignment.

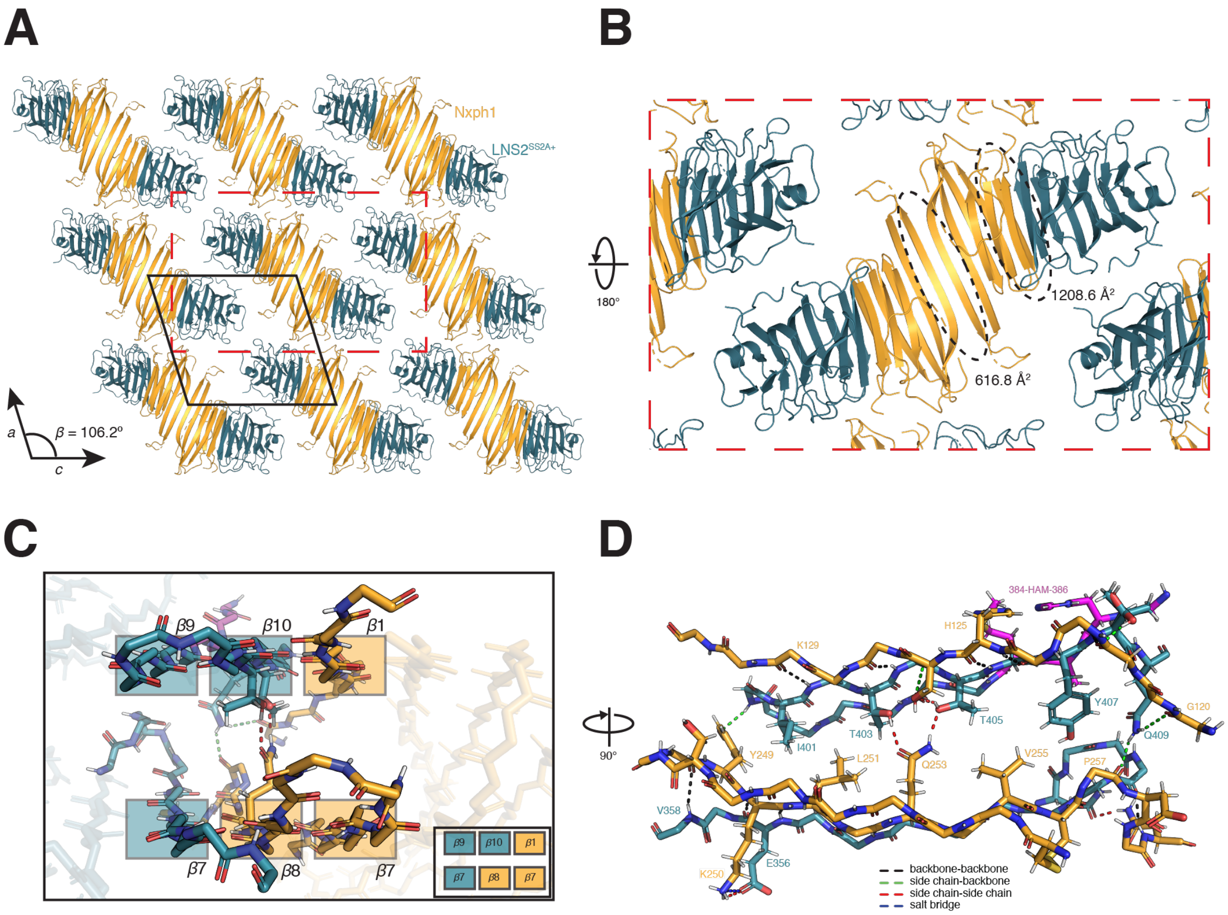


**Appendix Figure S3** Packing of complexes in Nxph1-LNS2$^{SS2A+}$ crystals and details of Nxph1-LNS2$^{SS2A+}$ interface architecture. (**A**) Selected *ac* plane of molecules in crystals of the Nxph1-LNS2$^{SS2A+}$ complex. The face of the *ac* plane is outlined in black. (**B**) Close-up view and 180° rotation of the section of the *ac* plane outlined in red in panel **A.** Of note are the large heterodimeric interfaces between Nxph1 and LNS2$^{SS2A+}$ and the smaller homodimeric interfaces between Nxph1 molecules. Interfaces are outlined with dotted ovals and buried surface areas of interfaces indicated. (**C**) Stick representation of β-strands in the complex interface. β-strands are labeled and highlighted. Polar interactions traversing the Nxph1-LNS2 β sandwich are shown as dotted lines. (**D**) Rotated view of the complex in **C,** with all detected polar interactions shown as dotted lines.

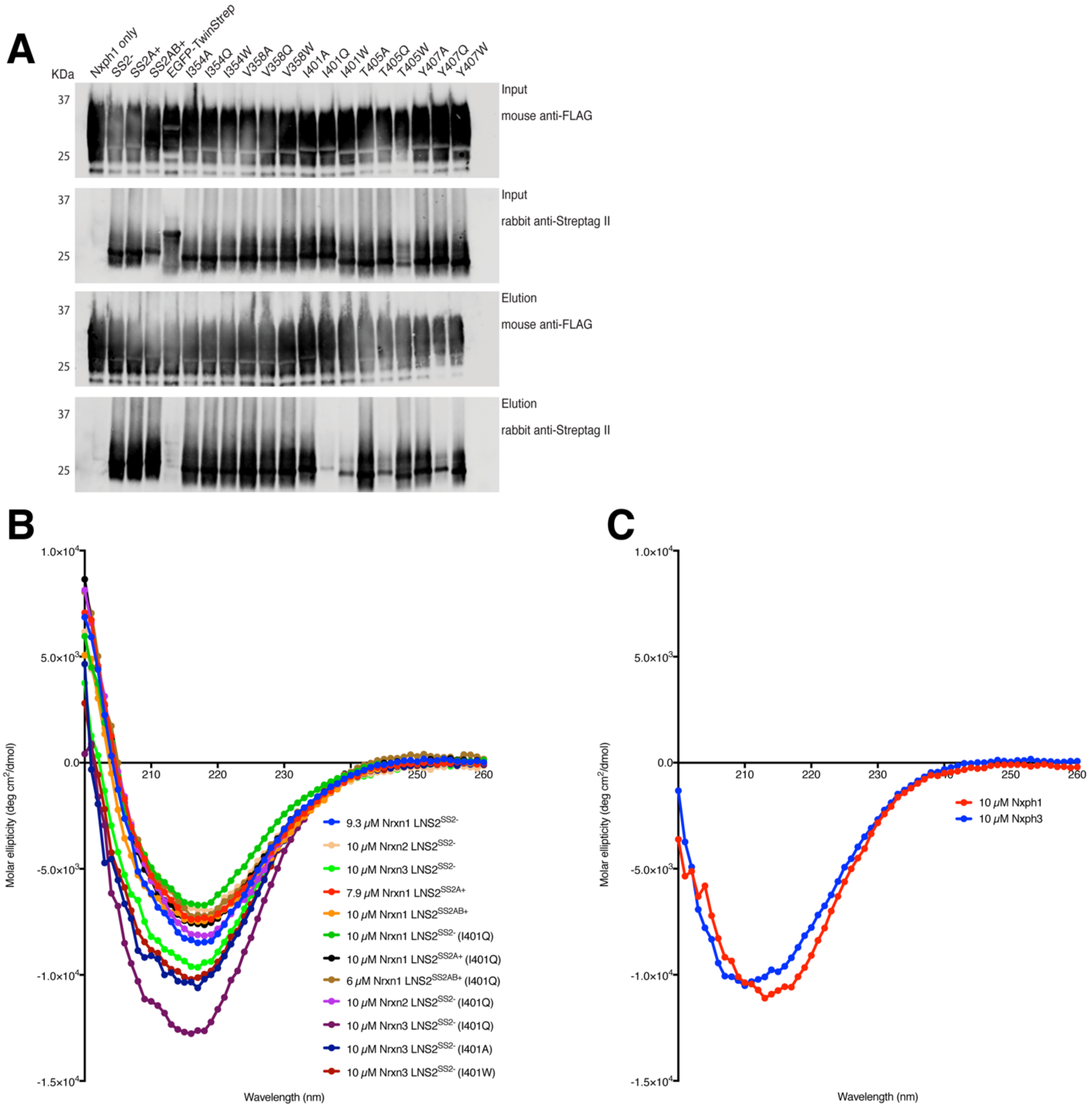


**Appendix Figure S4** Co-immunoprecipitation of Nrxn3 LNS2$^{SS2-}$ mutants with Nxph1 and Circular Dichroism spectra of LNS2 and Nxph proteins**.** (**A**) Nrxn3 LNS2-TwinStrep proteins were co-immunoprecipitated with Nxph1-2×FLAG-His using M2 anti-FLAG magnetic beads. (Mutated Nrxn3 residues are numbered at the top according to the corresponding residue numbers in the UniProt Nrxn1 sequence Q9CS84-1). EGFP-TwinStrep was used as a negative control. Inputs and elutions were probed with mouse anti-FLAG or rabbit anti-Streptag II and appropriate LI-COR secondary antibodies. (**B**) CD spectra of LNS2 proteins. (**C**) CD spectra of Nxph1 and Nxph3.

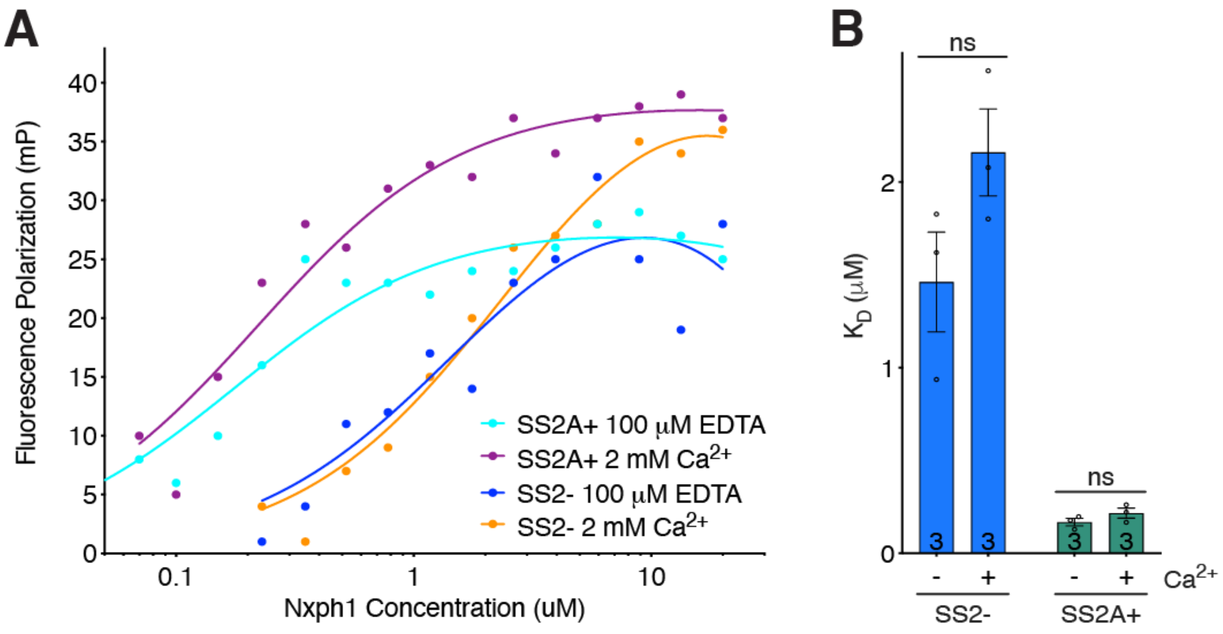


**Appendix Figure S5** Trace amounts of $Ca^{2+}$ in buffer do not affect the binding affinity of Nxph1 for Nrxn1 LNS2$^{SS2-}$ and Nrxn1 LNS2$^{SS2A+}$. (**A**) Representative binding curves are shown for LNS2$^{SS2-}$ and LNS2$^{SS2A+}$ interacting with Nxph1 in the presence of 2 mM $Ca^{2+}$ or 100 μM EDTA. (**B**) Comparison of binding affinities of Nxph1 for Nrxn1 LNS2$^{SS2-}$ and Nrxn1 LNS2$^{SS2A+}$ in the presence of 2 mM $Ca^{2+}$ or 100 μM EDTA. Replicate numbers are indicated in bars. Replicate numbers for experiments represented in **A** are shown in **B**. Error bars represent SEM and significance values were calculated using Welch's t-test.

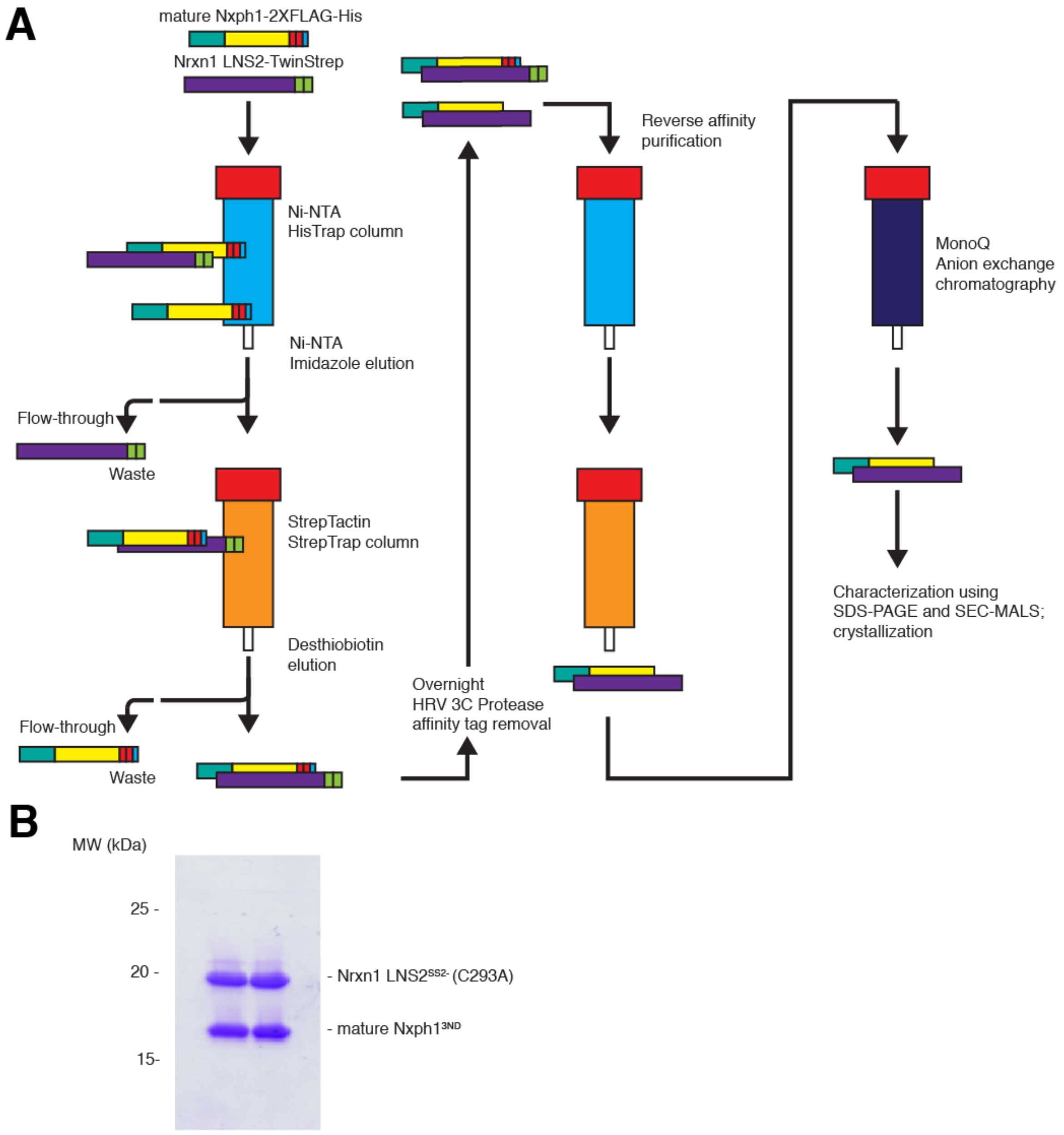


**Appendix Figure S6** Tandem affinity purification of Nxph1-Nrxn1 LNS2 complexes. (**A**) Nxph1-LNS2 complexes were co-expressed and secreted from HEK293S GnTI- cells using the BacMam expression system. His-tagged Nxph1 was co-purified from concentrated media in complex with TwinStrep-tagged LNS2 using tandem Ni-NTA-StrepTactin affinity chromatography. Affinity tags were removed with overnight HRV 3C protease digestion. Purified complexes were re-run over tandem Ni-NTA-StrepTactin columns to remove any complexes with un-cleaved affinity tags and further purified using anion exchange chromatography. (**B**) SDS-PAGE of peak anion exchange fractions of the Nxph1$^{3ND}$-LNS2$^{SS2-}$ (C293A) complex.

## Appendix References

Missler M & Südhof TC (1998) Neurexophilins form a conserved family of neuropeptide-like glycoproteins. *J. Neurosci.* **18:** 3630–3638

Sievers F, Wilm A, Dineen D, Gibson TJ, Karplus K, Li W, Lopez R, McWilliam H, Remmert M, Söding J, Thompson JD & Higgins DG (2011) Fast, scalable generation of high-quality protein multiple sequence alignments using Clustal Omega. *Mol. Syst. Biol.* **7: 539**
