# Supplementary material for: Structures of neurexophilin–neurexin complexes reveal a regulatory mechanism of alternative splicing
Source: EMBO J. 2019 Sep 30;38(22):e101603. doi: 10.15252/embj.2019101603 (PMC6856630; doi:10.15252/embj.2019101603)
Supplement: Supplementary file 2 — Expanded View Figures PDF [file EMBJ-38-e101603-s002.pdf]

Expanded View Figures

Figure EV1. Analysis of Nxph, LNS2, and Nxph1-LNS2 complexes by SEC-MALS.

A–H SEC-MALS traces for (A) Nrxn1 LNS2^{SS2-}, (B) Nrxn1 LNS2^{SS2A+}, (C) Nxph1^{3ND}-Nrxn1 LNS2^{SS2-} (C293A), (D) Nxph1^{3ND}-Nrxn1 LNS2^{SS2A+}, and (E) Nxph1-2XFLAG-His (from HEK293F cells). (F) Overlay of traces shown in panels (A–E), (G) Nxph3-2XFLAG-His (from HEK293F cells), (H) I401Q mutant of Nrxn2 LNS2^{SS2-}. The mean molecular weight (MW) of each protein is indicated near each peak. The poor fits of the molecular weight curves in (E and G) are due to oligomeric mixtures of Nxph1 and Nxph3 molecules, respectively.

© 2019 The Authors

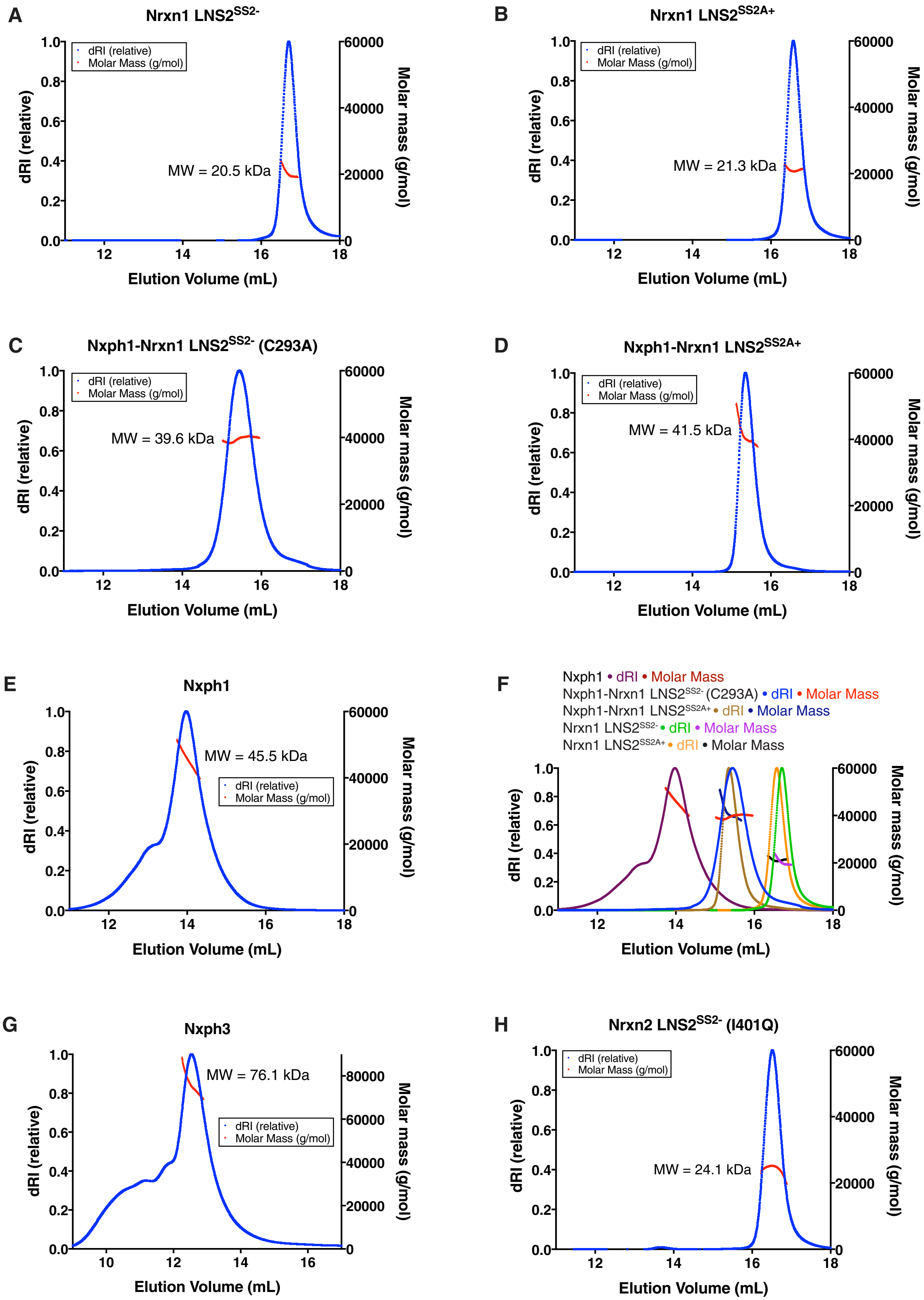

Figure EV1.

 © 2019 The Authors

Figure EV2. The Nrxn1 LNS2 Nxph1-binding site is highly conserved only in vertebrate neurexins and proximal to the SS2 insert site.
Sequence alignment of neurexin LNS2 domains from vertebrate and invertebrate species is shown. Sequences are numbered in reference to the canonical UniProt mouse Nrxn1 sequence Q9CS84-1. Secondary structure elements from LNS2^{SS2A+} in the Nxph1-LNS2^{SS2A+} structure are shown at the top. Fully conserved sequences are highlighted in red, semi-conserved sequences are highlighted in blue, and non-conserved sequences are highlighted in white. Inserts in SS2 are highlighted in magenta. Key Nxph1-binding residues are underlined. The disulfide bridge in Nrxn1 LNS2^{SS2A+} is shown as a red line. UniProt accession numbers for the sequences used are as follows: Mouse Nrxn1 (Q9CS84), Mouse Nrxn2 (E9PUM9), Mouse Nrxn3 (Q6P9K9), Human Nrxn1 (Q9ULB1), Human Nrxn2 (Q9P2S2), Human Nrxn3 (Q9Y4C0), Rat Nrxn1 (Q63372), Rat Nrxn2 (Q63374), Rat Nrxn3 (Q07310), Cow Nrxn1 (Q28146), Cow Nrxn2 (E1BFN9), Zebrafish Nrxn1 (A1XQX0), Zebrafish Nrxn2 (B7ZD56), Zebrafish Nrxn3 (A1XQX8), Frog Nrxn2 (F6YGH1), Frog Nrxn3 (F6WM05), Chicken Nrxn1 (Q9DDD0), Chicken Nrxn3 (D0PRN3), Sea slug Nrxn (G0WLR9), Ant Nrxn3 (F4WZ00), and Fruit fly Nrxn1 (Q9VCZ9). Note: The sequence for Mouse Nrxn3 SS2AB was taken from published coordinates (Schreiner *et al*, 2014) and manually inserted into the alignment.

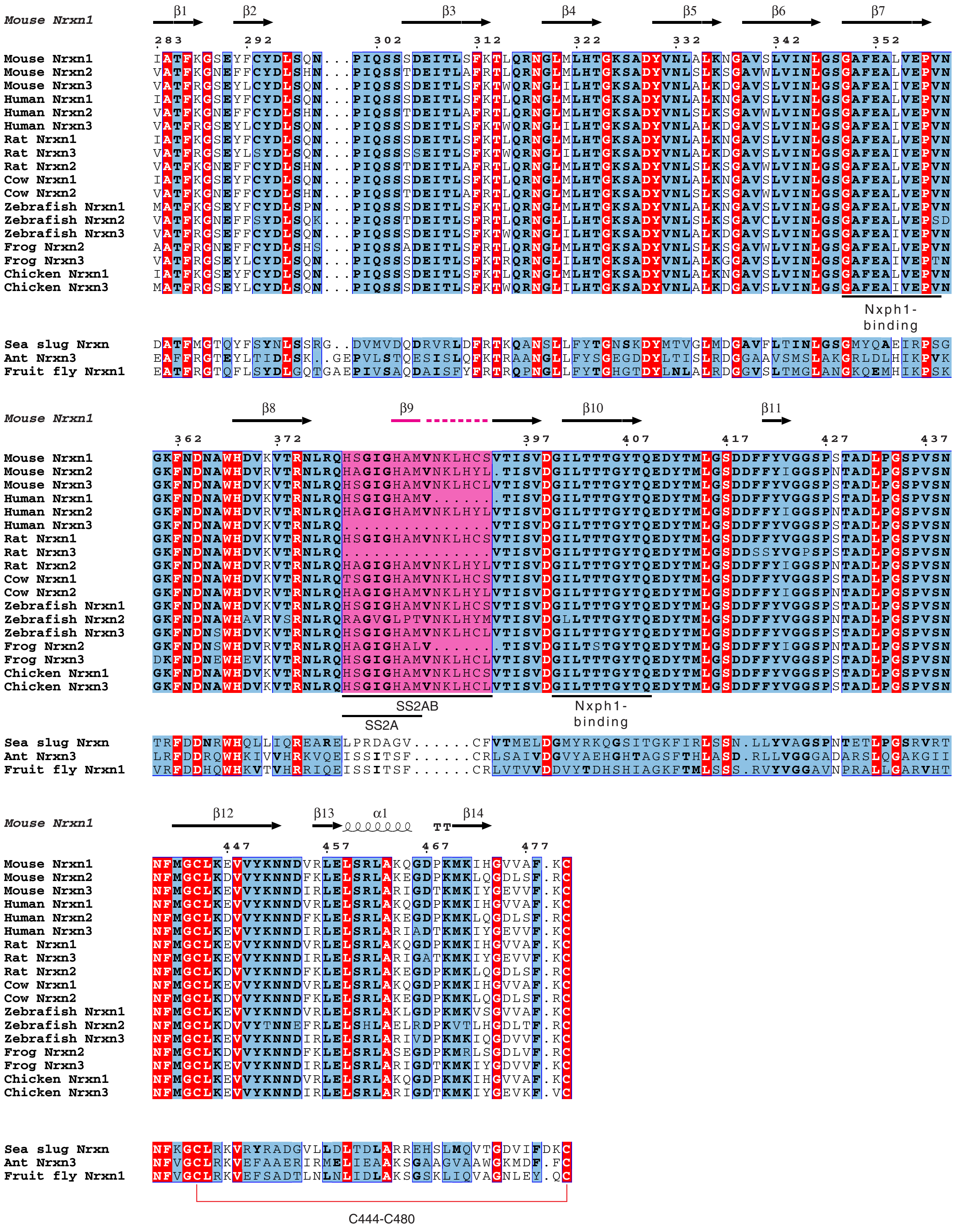

Figure EV2.

© 2019 The Authors

Figure EV3. The Nxph1 LNS2-binding site is highly conserved in vertebrate neurexophilins.

Sequence alignment of neurexophilins from vertebrates is shown. Secondary structure elements from Nxph1 in the Nxph1-LNS2^{SS2-} structure are shown at the top. This alignment only shows regions of neurexophilins aligned to mature Nxph1. Fully conserved sequences are highlighted in red; semi-conserved sequences are highlighted in yellow, and non-conserved sequences are highlighted in white. Key Nrxn1 LNS2-binding residues are underlined. Disulfide bridges are shown as orange lines. GenBank or RefSeq accession numbers for the sequences used are as follows: Rat Nxph1 (AAI05771.1), Rat Nxph2 (XP_008759839.2), Rat Nxph3 (AAI66603.1), Rat Nxph4 (AAH81805.1), Mouse Nxph1 (NP_032777.3), Mouse Nxph2 (EDL08154.1), Mouse Nxph3 (EDL15984.1), Mouse Nxph4 (EDL24512.1), Cow Nxph1 (AAX08911.1), Cow Nxph2 (NP_776831.1), Cow Nxph3 (NP_001179753.1), Cow Nxph4 (DAA29666.1), Human Nxph1 (BAE45730.1), Human Nxph2 (BAD11132.1), Human Nxph3 (AAQ88890.1), Human Nxph4 (AAQ88977.1), Frog Nxph1 (NP_001120374.1), Frog Nxph3 (NP_001096477.1), Zebrafish Nxph1 (AAH76424.1), Zebrafish Nxph2 (XP_698522.2), Zebrafish Nxph3 (XP_691473.2).

© 2019 The Authors

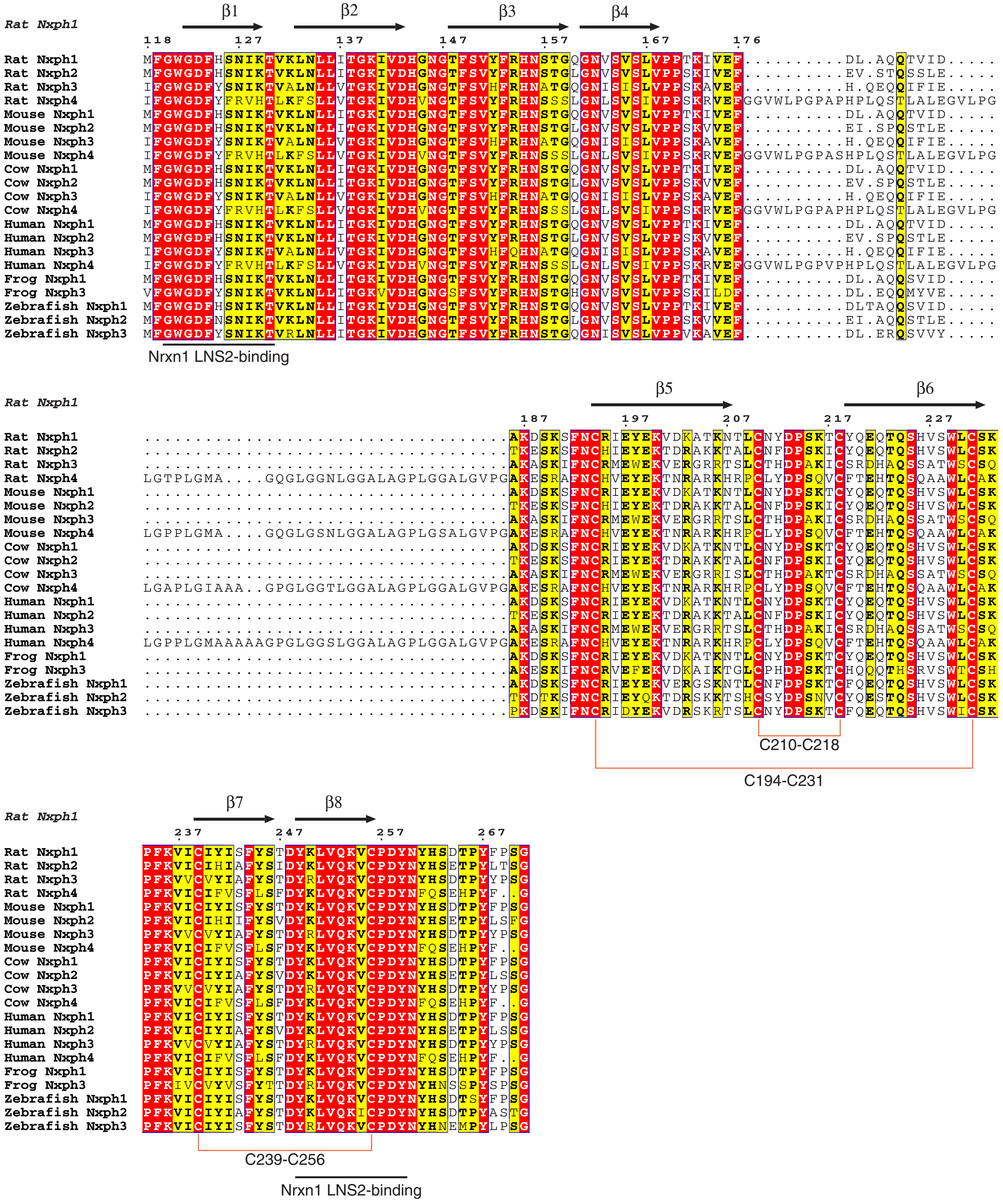

Figure EV3.

 © 2019 The Authors

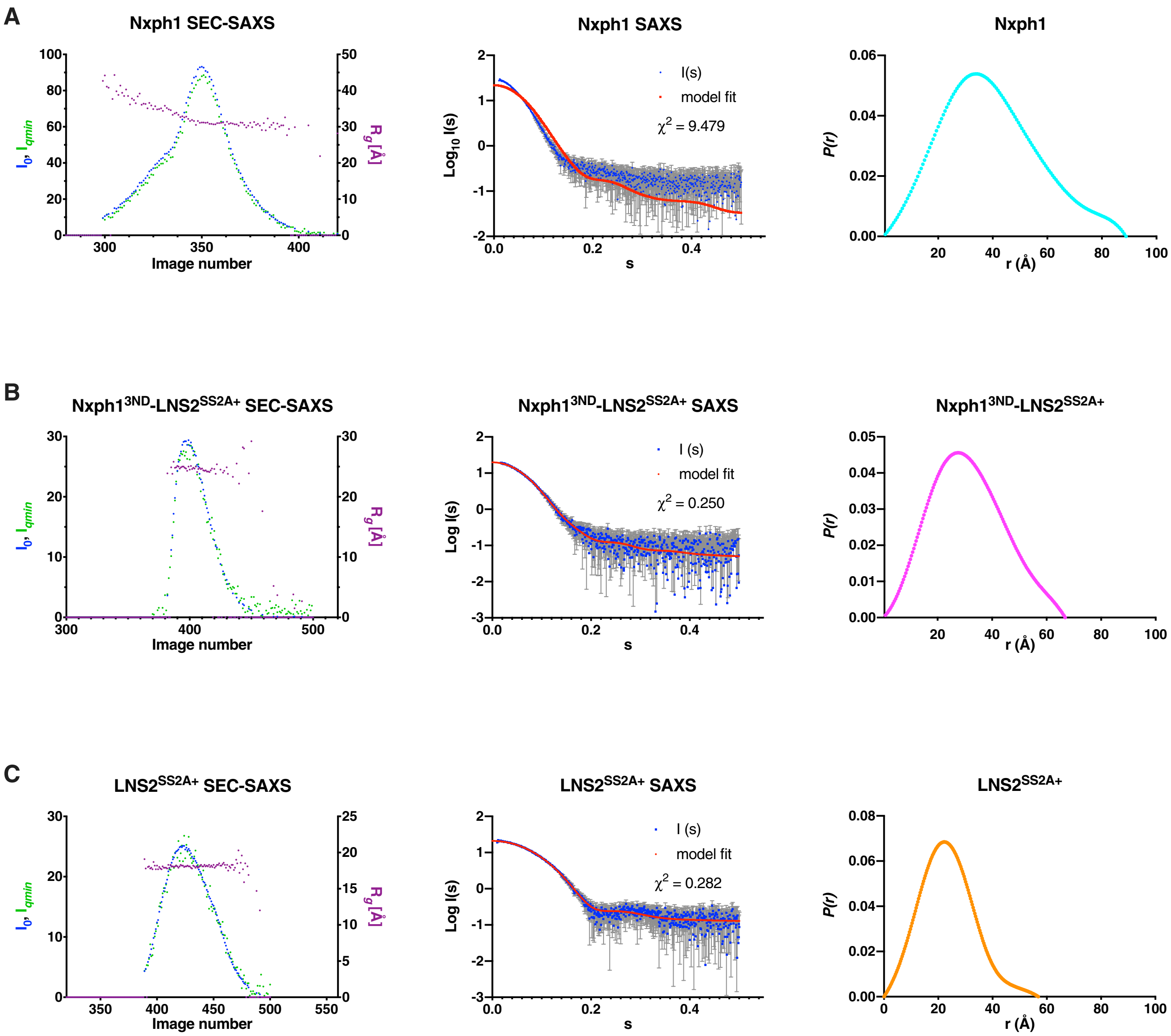

Figure EV4. SEC-SAXS analysis of Nxph1 and Nrxn1 LNS2 alone and in complex.

A–C SEC-SAXS data for (A) Nxph1, (B) Nxph1^{3ND}-Nrxn1 LNS2^{SS2A+}, and (C) LNS2^{SS2A+}. For each sample, SEC-SAXS traces are shown along with peak SAXS data with crystallographic model fit and pair-wise distance distribution functions. Scattering data represent the average of five technical replicates, and error bars in the middle panels represent SD.

Figure EV5. The I401Q mutation reduces the affinity of Nxph-Nrxn1 LNS2^{SS2-} interactions.

A Confocal images of co-expressed wild-type and I401Q mutant Nrxn1 LNS2^{SS2-} with Nxph1–4. Scale bar: 50 μm.
B BLI data showing reduced binding affinity of Nxph1 and Nxph3 to Nrxn1–3 LNS2^{SS2-} (I401Q) mutants. Error bars represent SEM, and replicate numbers are indicated in bars. Significance values were calculated using Welch's *t*-test, *$P < 0.05$, **$P < 0.01$, ***$P < 0.001$.

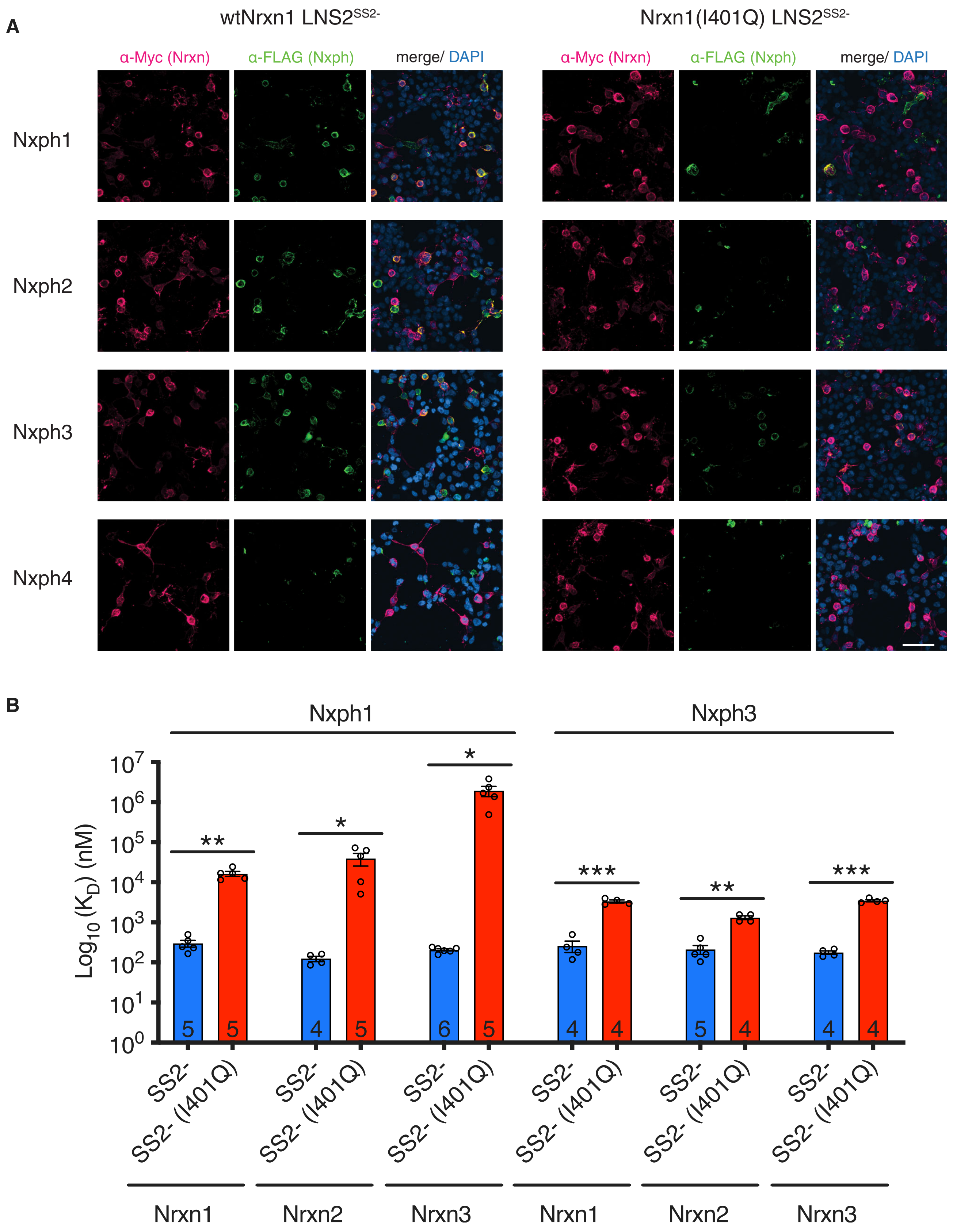

Figure EV5.

© 2019 The Authors
