# Supplementary material for: Structures of neurexophilin–neurexin complexes reveal a regulatory mechanism of alternative splicing
Source: EMBO J. 2019 Sep 30;38(22):e101603. doi: 10.15252/embj.2019101603 (PMC6856630; doi:10.15252/embj.2019101603)
Supplement: Supplementary file 3 — Table EV1 [file EMBJ-38-e101603-s003.docx]

**Table EV1 – Molecular weights calculated from SEC-SAXS data**

| SEC-SAXS sample | Calculated molecular weight  (predicted monomer MW) kDa |
| --- | --- |
| Major Nxph1 peak | 58.15 (29) |
| High molecular weight Nxph1 shoulder | 124.45 (29) |
| Nxph1^3ND^-LNS2^SS2A+^ peak | 41.08 (41) |
| LNS2^SS2A+^ peak | 18.68 |

The calculated molecular weights in the table were determined using the Bayesian Inference method in ATSAS.
